# Nosocomial transmission of extensively drug resistant *Acinetobacter baumannii* strains in a tertiary level hospital

**DOI:** 10.1371/journal.pone.0231829

**Published:** 2020-04-17

**Authors:** Paúl Alexis López-Durán, Salvador Fonseca-Coronado, Lucila Maritza Lozano-Trenado, Sergio Araujo-Betanzos, Deniria Alejandra Rugerio-Trujillo, Gilberto Vaughan, Elsa Saldaña-Rivera

**Affiliations:** 1 Departamento de Bioinformática y Biotecnología Genómica, Escuela Nacional de Ciencias Biológicas, Instituto Politécnico Nacional, Ciudad de México, México; 2 Laboratorio de Investigación en Enfermedades Infecciosas; Unidad de Investigación Multidisciplinaria, Facultad de Estudios Superiores Cuautitlán, Universidad Nacional Autónoma de México, México City, Edo. de México, México; 3 Facultad de Ciencias de la Salud, Universidad Anáhuac, Campus Norte, Naucalpan de Juárez, Estado de México, México; 4 Escuela Militar de Graduados de Sanidad, Secretaría de la Defensa Nacional, Ciudad de México, México; 5 Hospital Central Militar, Secretaría de la Defensa Nacional, Ciudad de México, México; Northwestern University Feinberg School of Medicine, UNITED STATES

## Abstract

*Acinetobacter baumannii* is an opportunistic infectious agent that affects primarily immunocompromised individuals. *A*. *baumannii* is highly prevalent in hospital settings being commonly associated with nosocomial transmission and drug resistance. Here, we report the identification and genetic characterization of *A*. *baumannii* strains among patients in a tertiary level hospital in Mexico. Whole genome sequencing analysis was performed to establish their genetic relationship and drug resistance mutations profile. Ten genetically different, extensively drug resistant strains were identified circulating among seven wards. The genetic profiles showed resistance primarily against aminoglycosides and beta-lactam antibiotics. Importantly, no mutants conferring resistance to colistin were observed. The results highlight the importance of implementing robust classification schemes for advanced genetic characterization of *A*. *baumannii* clinical isolates and simultaneous detection of drug resistance markers for adequate patient’s management in clinical settings.

## Introduction

*Acinetobacter baumannii* is a Gram negative coccobacillus that affects primarily immunocompromised individuals. Patients undergoing intensive care, emergency surgery, mechanical ventilation, tracheal intubation, central vein catheterization, urinary catheter, continuous renal replacement therapy, and other invasive procedures are at increased risk of infection [1–3].

The World Health Organization lists the carbapenem resistant *A*. *baumannii* on the pathogen critical priority list [4], making it an increasingly growing public health problem worldwide. Multidrug (MDR) resistance among *A*. *baumannii* strains is driven by rapid gene mutations or transfer of exogenous resistance genes by mobile genetic elements, such as plasmids, transposons or insertion sequences. Resistance to beta-lactam and aminoglycosides is also a rapidly growing public health problem globally. Likewise, circulation of carbapenem-resistant *A*. *baumannii* strains aggravates the antibiotic resistance scenery, making patient’s management challenging [5]. As a result, occurrence of transmission networks associated with MDR in clinical settings has become a growing health issue in different regions of the world [6]. MDR refers to strains that exhibit resistance to more than three or more antimicrobial drug classes, while extensively drug resistant (XDR) strains display resistance to all but two drug classes [7]. Pan-drug resistance (PDR) indicates resistance to all drug classes including carbapenems, colistin, and other polymyxins [8].

Here, we describe the circulation of ten genetically distinguishable strains among patients in seven wards in a third level hospital. Timely identification of transmission networks of multidrug resistant strains is of the utmost importance to prevent the spread of highly virulent bacteria and appropriate patient’s management.

## Methods

### Bacterial isolates and susceptibility testing

Ten isolates were obtained from patients hospitalized at the “Hospital Central Militar” in Mexico City collected between May and November of 2018. A written informed consent was obtained from each patient. The study was approved by the Research Ethics Committee at Hospital Central Militar (approval no. 045/2017). Patients for whom sample isolates were identified as *Acinetobacter spp*. were included in the study. Demographic and clinical data is listed on Table 1.

**Table 1:** Patients’ clinical characteristics.

ID	Age	Gender	Diagnosis	Ward	Sample type	Collection date 2018
P001	63	M	2nd degree burn wound	Oncology and Restructuring Surgery	Wound secretion	July 12
P003	65	F	Inguinal hernioplasty	Women's Surgery	Bronchoaspirate	June 8
P004	56	M	Nephrectomy	Urology	Abscesses	June 12
P006	30	M	Severe head trauma	Neurosurgery	Total blood	June 11
P007	46	F	Acute alithiasic cholecystitis	Intensive care unit	Urine	May 17
P008	63	F	Acute pancreatitis	Women surgery	Pleural fluid	May 16
P009	58	F	Septic shock	Adult Intensive Therapy Unit	Bronchoaspirate	August 14
P010	64	F	Craniectomy	Nephrology	Total blood	September 12
P011	71	M	Hyperplasia of the prostate	Adult Intensive Therapy Unit	Bronchoaspirate	October 5
P012	50	M	Epilepsy	Neurology	Urine	November 11

Bacterial identification and antibiotic minimum inhibitory concentration (MIC) testing were performed using the Vitek 2 Compact system (bioMérieux, Basingstoke, UK) following the manufacturer's instructions.

### Isolation of DNA

*A*. *baumannii* genomic DNA was isolated and purified using the QIAamp DNA Mini Kit (Qiagen) following the manufacturer’s recommendations. The Quant-iT Pico Green dsDNA Reagent (Invitrogen, USA) was used to determine DNA concentration. Fluorescence was quantified using the Hidex Sense Microplate Reader. Final DNA concentration was adjusted at 0.2 ng/μl.

### Whole genome sequencing (WGS)

Total DNA was used to perform sequencing on a MiSeq instrument (Illumina, San Diego, CA) using 2x300 Nextera XT library preparation kit (Illumina, San Diego, CA), following the manufacturer’s instructions. Average library sizes were evaluated using a High Sensitivity DNA kit on the Agilent 2100 Bioanalyzer according to the manufacturer’s instructions. The obtained files are available at the NCBI SRA database under BioProject ID PRJNA587517. Quality of sequencing reads was determined using FASTQC version 0.11.8 [9]. Reads of poor quality, adapter sequences removal as well as sizing was performed using Trimmomatic version 0.39 [10]. FASTQC was used to evaluate quality scores before trimming. Kmergenie version 1.7051 [11] was used to determine the best kmer for genome assembly. All sequences were assembled using SPAdes version 3.13.0 [12] and ABySS version 2.1.5 [13]. QUAST was used to derive assembly metrics including N50, L50 and number of contigs [14]. All sequences were annotated with PROKKA version 1.13.7 [15]. KmerFinder 3.1 was used to determine the species (https://cge.cbs.dtu.dk/services/KmerFinder/) [16].

### Multilocus sequencing typing (MLST)

All loci (gltA, gyrB, gdhB, recA, cpn60, gpi, rpoD) included in the Oxford and Pasteur schemes (cpn60, fusA, gltA, pyrG, recA, rplB and rpoB) were derived from WGS originated from all ten isolates. Clustering was performed using UPGMA dendrograms as implemented in the Orange suite (https://orange.biolab.si).

### Core genome multilocus sequencing typing (cgMLST)

Allele calling was performed using the chewBBACA algorithm [17], with the corresponding *A*. *baumannii* training set (https://github.com/mickaelsilva/prodigal_training_files). Additionally, ten *A*. *baumannii* strains (GenBank accession numbers CP014528.1, CP018254.1, CP035672.1, CP027611.1, CP021326.1, CP033869.1, CP040259.1, CP041035.1, CP046654.1, CP009257.1) were used as reference sequences. The cgMLST analysis included a total of 2390 alleles across the genome. Tree visualization was carried out with PHYLOViZ [18].

### Characterization of drug resistant mutations

Characterization of resistance-mutations was performed using ResFinder v3.1.0 [19].

## Results

### Clinical data

Samples were collected between May and November 2018 from patients hospitalized for more than five days at seven different wards. Patients’ clinical information is shown in Table 1.

### Genetic relatedness

**Fig 1:**
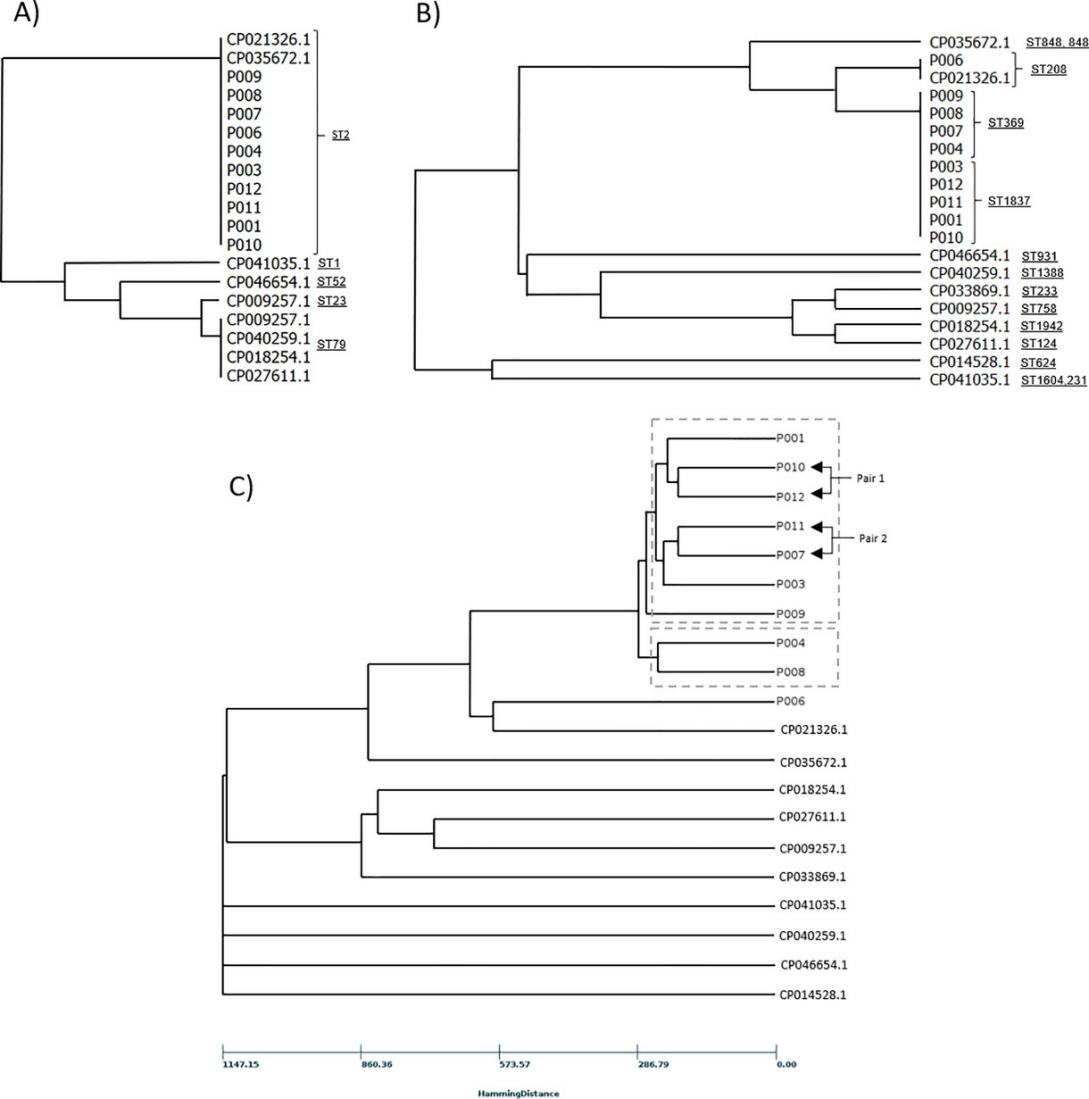
Genetic analysis of *Acinetobacter baumannii* strains. WGS were used to extract all loci required for Pasteur, Oxford schemes and cgMLST approach. The corresponding dendrograms were generated based on genetic distances among isolates and reference sequences. A) Pasteur scheme classified all isolates belonging to one distinctive sequence type (ST2). B) Oxford scheme grouped all outbreak sequences into two different sequence types (ST369 and ST1837). The unrelated sample derived from patient P006 was classified as ST208 along with reference strain CP021326.1. All other unrelated reference strains were classified as different ST. C) The cgMLST analysis showed a main branch with two distinctive clusters. Samples P004 and P008 formed a pair included in the small cluster while two pair of sequences (P010:P012 and P011:P007) were included in the major cluster along with samples P001, P003 and P009. Unrelates train P006 remained genetically distant from all other clinical samples.

All ten isolates were subjected to WGS and analyzed for genetic relatedness and drug resistance profile. The MLST analysis according to Pasteur scheme showed that all strains belonged to ST2 (Fig 1A). Additionally, two unrelated reference strains (CP021326.1 and CP035672.1) were also classified as ST2. All other reference strains belonged to other distinctive ST.

The Oxford scheme classified the outbreak strains into two different types. Samples P004, P007, P008 and P009 belonged to ST369 while samples P001, P003, P010, P011 and P012 were classified as ST1837 (Fig 1B). Additionally, samples P006 and reference strain CP021326.1 grouped together as ST208. Sample P006 was recovered from a patient originally diagnosed with *A*. *baumannii* infection in a hospital in Tijuana, Baja California, who was subsequently referred to the Hospital Central Militar in Mexico City. Thus, this isolate was expected, to a certain degree, to remain genetically distant from the main cluster identified in our hospital.

cgMLST analysis using WGS was able to discriminate strains from each other with better resolution. All samples grouped together in one branch exhibiting two distinctive clusters. Cluster 1 included two sample pairs (P010:P012 and P011:P007) besides samples P001, P003 and P009 (Fig 1C). Cluster 2 include a pair composed by samples P004 and P008. All samples were closely genetically related but different with the exception of sample P006. Sample P006 was not part of any of the clusters in the main branch. This was expected since this patient was infected by a different source in a different geographical region, and therefore unrelated to all other cases. Distance Matrix between all isolates corroborated the phylogenetic findings (S1 Fig).

### Antibiotic susceptibility profiles

**Fig 2:**
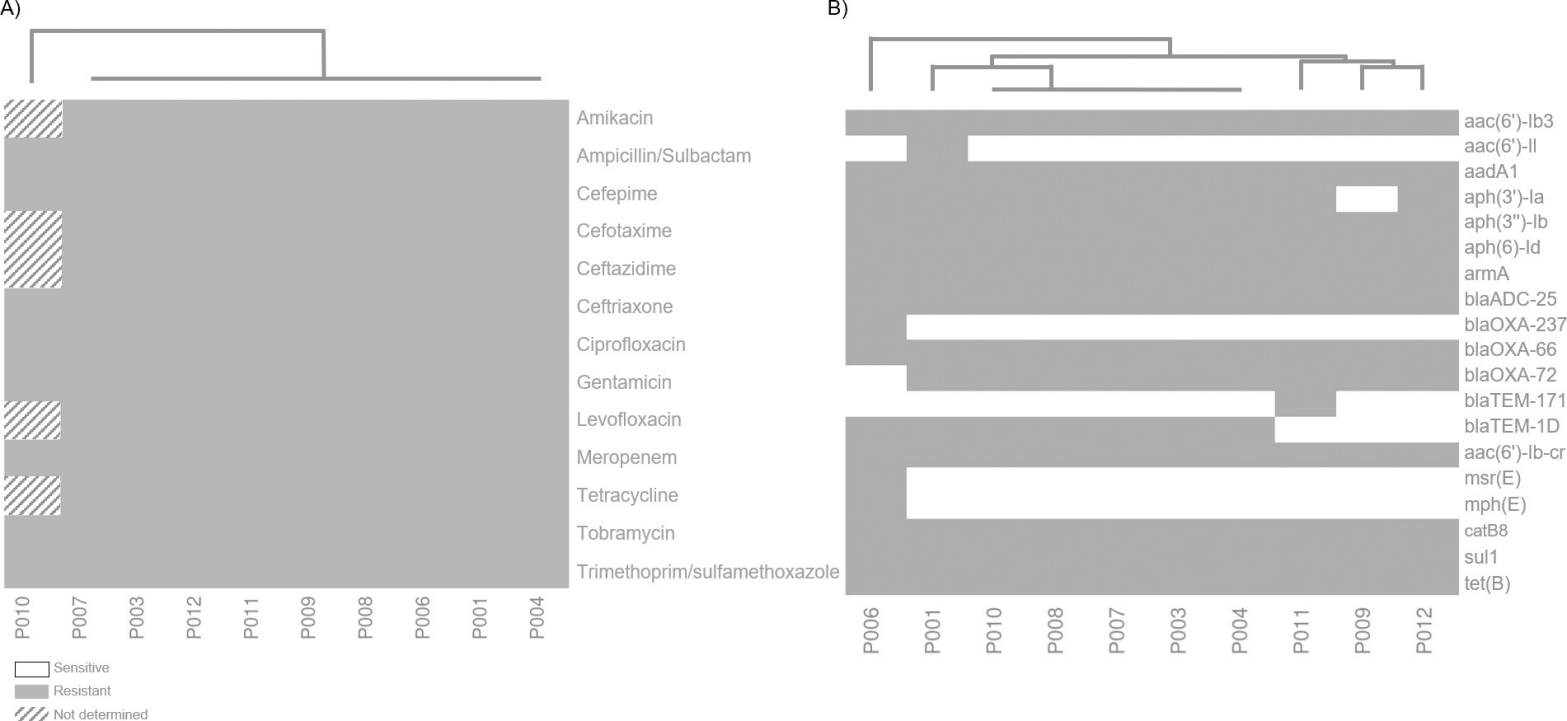
Antibiotic resistance profile. A) All strains were subjected to antibiotic resistance testing using amikacin, ampicillin/sulbactam, cefepime, cefotaxime, ceftazidime, ceftriaxone, ciprofloxacin, gentamicin, levofloxacin, meropenem, tetracycline, tobramycin and trimethoprim/sulfamethoxazole. Two distinctive patterns were observed. All samples exhibited the same profile. Sample P010 had an incomplete antibiotic resistance testing; and therefore, could not be classified accordingly. B) Drug resistance mutation detection was inferred from WGS. Six distinctive patterns were identified.

The MIC method displayed a high degree of drug resistance among these isolates. All ten strains were classified as XDR according to the scheme by Magiorakos et al. in 2012 [20]. All strains displayed resistance to penicillin’s, cephalosporins, carbapenems, fluoroquinolones, aminoglycosides, and sulfonamides. The complete susceptibility pattern is shown in Fig 2A.

Likewise, the corresponding sequence analysis confirm the presence of drug resistance mutations confirming the XDR nature of these strains. The main antibiotic resistance genes identified among these isolates were associated with aminoglycosides (genes: *aac(6')-*Ib3, *aac(6')-*Il, *aad*A1, *aph(3')-*Ia, *aph(3'')-*Ib, *aph(6)-*Id, *arm*A) and beta-lactam antibiotics (*bla*_ADC-25_, *bla*_OXA-237_, *bla*_OXA-66_, *bla*_OXA-72_, *bla*_TEM-171_, *bla*_TEM-1D_). In addition, mutations associated with resistance to fluoroquinolones (*aac(6')-*Ib-cr), MLS—macrolide, lincosamide and streptogramin B (*msr*(E), *mph*(E)), phenicol (*cat*B8), sulfonamides (*sul*1) and tetracycline (*tet*(B)) were also identified, although with less frequency (Fig 2B). Importantly, no mutations associated with colistin resistance was identified.

## Discussion

Here we describe the molecular characterization and drug resistance profiles of ten XDR *A*. *baumannii* strains associated with nosocomial transmission in a tertiary hospital, the data presented here is not representative of the molecular epidemiology of *A*. *baumannii* in the country. As reported previously, it has been observed that *A*. *baumannii* is transmitted primarily by contact or invasive procedures, such as surgery, mechanical respirator or tracheostomy [6,21]. It has been also described that burned patients are particularly susceptible to an *Acinetobacter* infection during hospitalization [22]. All patients enrolled in this study underwent an invasive procedure and/or were immunocompromised.

The extensive drug resistance profile displayed by the analyzed strains showed a remarkable resistance to primarily aminoglycosides and β-lactam antibiotics. However, resistance to fluoroquinolones, phenicol, sulfonamides, tetracycline was also commonly observed among the isolates. Less frequent was the identification of mutants conferring resistance to macrolides/lincosamides and streptogramin. Although the P010 antibiogram is not complete, it can be inferred by genetic analysis that its profile is very similar to the other strains. This resistance pattern among XDR strains has been previously described in other countries [23,24]. Different studies have reported the circulation, and corresponding transmission of *A*. *baumannii* strains in other clinical settings in Mexico [1,25,26]. However, and to the best of our knowledge, this is the first report describing the circulation of XDR strains in the country.

*A*. *baumannii* infections that are resistant to carbapenems have been associated with high mortality rates. The most important mechanism in A. baumannii for carbapenem resistance is the production of carbapenem-hydrolyzing class D OXA-type β-lactamases [27]. Of note, most of the strains are carrying mutations associated with resistance in blaOXA-66 and blaOXA-72.

The blaOXA-66 is the gene that encode for the most frequent carbapenemase in the world, coexisting with others carbapenemases just like the blaOXA-23 and blaNDM-1 genes [28]. blaOXA-72 is the main allelic variant within the blaOXA24/40 group, and strains producing OXA-72 has previously been reported in hospitals and other sources in Eastern Europe, Southern Asia and South America [27]. Special attention must be point out on this element that represent a flexible way to transfer carbapenem resistance.

Regarding the phylogenetic classification, in our study Pasteur scheme showed no diversity in the strains being all assigned to the sequence type 2, it has largely showed that this scheme exhibits less discerning capacity among closely related strains [29]. According to the Oxford Scheme, two clusters of transmission were observed among patients sharing XDR *A*. *baumannii* strains (ST1837 y ST369), The ST183 and ST369 has been reported at other hospitals in Mexico, the regional Hospital Ignacio Zaragoza, Mexico City (n = 18), the Dr. José Eleuterio González University Hospital, Monterrey (n = 13) and the Hospital Central Monterrey (n = 1) [1,25,26]. About the foreign strain ST208, this has been reported in the Nuevo Leon University Hospital [26].

WGS analysis showed the circulation of closely genetically related strains. However, no clonal transmission was identified in this study. This could be explained by multiple sources of infections in this setting. Similarly, no common procedure or direct contact between cases was reported. The enrolling of new cases is ongoing, and the corresponding clinical investigation is expected to be continued. It is noteworthy that other conventional typing approaches for *A*. *baumannii* did not provide sufficient resolution to accurately characterized these isolates. Analyses for both, the Oxford and Pasteur typing schemes, were carried out using loci extracted from WGS. Neither typing approach resulted in sufficient discrimination among these isolates to carry out the clinical investigation.

Implementation of WGS as advanced molecular characterization of infectious agents in tertiary hospitals could improve both, identification and genetic characterization including resistance profiles, facilitating outbreak investigations and molecular surveillance. This information will likely provide insights on the molecular epidemiology and routes of transmission exploited by different infectious agents in clinical settings.

## Supporting information

S1 Fig (TIFF)
