# Supplementary figures and images for: Nosocomial transmission of extensively drug resistant Acinetobacter baumannii strains in a tertiary level hospital
Source: PLoS One. 2020 Apr 17;15(4):e0231829. doi: 10.1371/journal.pone.0231829 (PMC7164640; doi:10.1371/journal.pone.0231829)

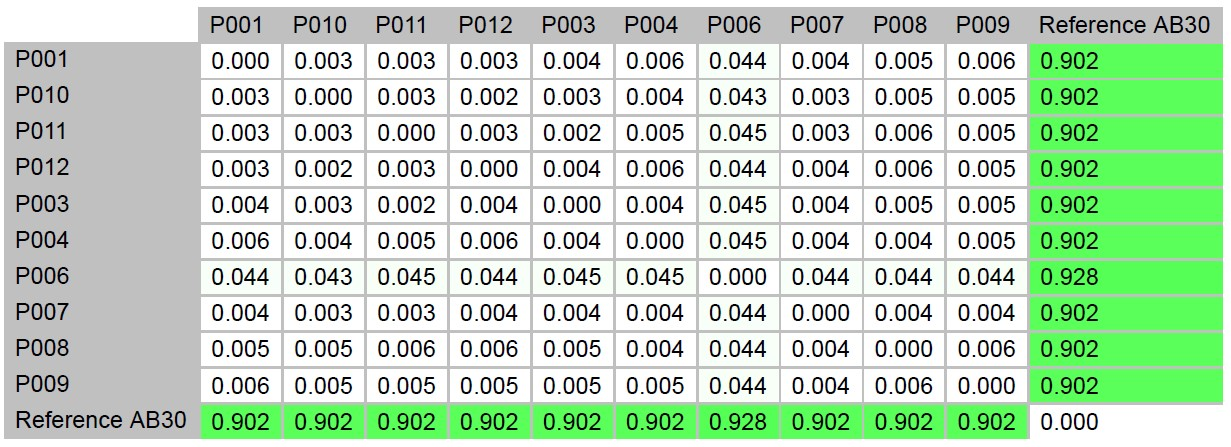

Supplement: S1 Fig — (TIFF) [file pone.0231829.s001.tiff]
